# Muscarinic Receptor-Dependent Long Term Depression in the Perirhinal Cortex and Recognition Memory are Impaired in the rTg4510 Mouse Model of Tauopathy

**DOI:** 10.1007/s11064-018-2487-x

**Published:** 2018-02-26

**Authors:** Sarah E. Scullion, Gareth R. I. Barker, E. Clea Warburton, Andrew D. Randall, Jonathan T. Brown

**Affiliations:** 1 School of Physiology and Pharmacology and Neuroscience, Medical Sciences Building, University of Bristol, Bristol, BS8 1TD UK; 2 Institute of Biomedical and Clinical Sciences, University of Exeter Medical School, University of Exeter, Hatherly Laboratories, Prince of Wales Road, Exeter, EX4 4PS UK

**Keywords:** Fronto-temporal dementia, Alzheimer’s disease, Synaptic plasticity, rTg4510

## Abstract

Neurodegenerative diseases affecting cognitive dysfunction, such as Alzheimer’s disease and fronto-temporal dementia, are often associated impairments in the visual recognition memory system. Recent evidence suggests that synaptic plasticity, in particular long term depression (LTD), in the perirhinal cortex (PRh) is a critical cellular mechanism underlying recognition memory. In this study, we have examined novel object recognition and PRh LTD in rTg4510 mice, which transgenically overexpress tau_P301L_. We found that 8–9 month old rTg4510 mice had significant deficits in long- but not short-term novel object recognition memory. Furthermore, we also established that PRh slices prepared from rTg4510 mice, unlike those prepared from wildtype littermates, could not support a muscarinic acetylcholine receptor-dependent form of LTD, induced by a 5 Hz stimulation protocol. In contrast, bath application of the muscarinic agonist carbachol induced a form of chemical LTD in both WT and rTg4510 slices. Finally, when rTg4510 slices were preincubated with the acetylcholinesterase inhibitor donepezil, the 5 Hz stimulation protocol was capable of inducing significant levels of LTD. These data suggest that dysfunctional cholinergic innervation of the PRh of rTg4510 mice, results in deficits in synaptic LTD which may contribute to aberrant recognition memory in this rodent model of tauopathy.

## Introduction

Dementias such as Alzheimer’s disease (AD) and fronto-temporal dementia (FTD) are characterised by severe cognitive impairment, including a spectrum of varied memory disorders. In particular, various forms of episodic and recognition memory are compromised, including visual recognition memory [1, 2]. Without doubt the substantial cortical atrophy associated with these forms of dementia will in time contribute to progressive deficits in visual recognition and other forms of memory. However, significant functional decline can appear prior to gross neurodegeneration [1]. This has been reinforced by recent a study characterizing cognitively “normal” middle-aged individuals with familial history of Alzheimer’s disease who exhibited lower accuracy on a perirhinal-associated task despite lacking perirhinal atrophy in MR imaging [3]. Such data indicate more subtle cell or network-level mechanisms may, therefore, underlie the visual recognition memory disturbances observed in dementia, at least in early disease stages. A concept that is increasingly supported by studies in animal models of dementia pathology (see below).

The perirhinal cortex (PRh) has been strongly linked to visual recognition memory [4, 5]. Anatomical lesions of the PRh result in an abolition of novel object discrimination (a behavioural model of visual recognition memory) in both rodents [6–9] and primates [10, 11]. Similar outcomes are observed during pharmacological blockade of glutamatergic synaptic transmission and plasticity in the PRh [12–14]. Furthermore, novel object discrimination can also be blocked by PRh infusions of pharmacological agents which inhibit synaptic plasticity. In this regard, both NMDA receptor antagonists, such as AP5 [13, 14], and muscarinic acetylcholine receptor (mAChR) antagonists, such as scopolamine interfere with novelty discrimination in rodents [15]. Interestingly, both AP5 and scopolamine have been shown to block long-term depression (LTD) of synaptic transmission in the PRh [15, 16]. These studies and others [17, 18] have led to the proposal that LTD in the PRh represents a pivotal synaptic basis of visual recognition memory.

In addition to the amyloid pathology associated with AD, patients suffering from a wide variety of forms of dementia (including AD and FTD) have extensive intracellular neuronal inclusions known as neurofibrillary tangles. These consist of aggregates of the microtubule-associated protein tau in a hyperphosphorylated state [19–21]. In contrast to APP-based mouse models of dementia, there are relatively few studies of synaptic plasticity in tau-based mouse models of dementia. The rTg4510 model of tauopathy overexpresses the P301L mutant form of tau found in fronto-temporal dementia with Parkinsonism associated with chromosome 17, in a regulatable, forebrain-restricted fashion [22]. A 2010 study reported that this model has an extensive synaptic pathology in the hippocampus, coupled with deficits in long term potentiation (LTP) of synaptic transmission in the CA1 region of the hippocampus [23]. Our own work in this model has suggested that LTP in the temporoamonic input from entorhinal cortex (ECtx) is affected more than Schaffer collateral LTP [24] something that may reflect the various neurophysiological changes observed in ECtx [25]. These synaptic deficits may in part underlie the reported impairment in spatial learning in this model [22, 24, 26]. We have also recently observed neurophysiological changes associated with the presence of a disease-associated C-terminal tau fragment [27].

In contrast to hippocampal-dependent learning and memory processes, there is a relative paucity of information regarding non-hippocampal learning in murine tauopathy models. In heterozygous P301S tau mice visual object recognition has been reported to be lost at 7, but not 3, months of age, an outcome that can be mimicked with viral delivery of a P310S tau construct to the perirhinal area. In the former model basal synaptic transmission was depressed but perirhinal LTD appeared normal. Interestingly, these workers find the effects of tauopathy on recognition memory and synaptic transmission can be rescued by manipulation of the perineuronal net [28, 29]. In this study we have examined novel object discrimination in male 8–9 month old rTg4510 mice a widely studied model in which the human disease-associated P301L tau species is overexpressed selectively in the mouse forebrain. We have also examined a form of mAChR-dependent LTD in the PRh, which is thought to underlie visual recognition memory.

## Materials and Methods

### Animals

All experiments used male adult (8–10 months) rTg(_tauP301L_)4510 that expressed the P301L tau mutation (4R0N) that is associated with frontotemporal dementia and parkinsonism linked to chromosome 17. The transgene was linked to a Ca^2+^ calmodulin kinase II promoter which drives forebrain specific over-expression of P301L tau. This mutant tau accumulates in an age-dependent manner, leading to spatial reference memory deficits from 4 months old and gross forebrain atrophy with predominant cell loss in CA1 hippocampal neurons from 8 months [22, 26]. These animals were compared with age-matched littermate wild-type (WT) control mice. The animals were singly or doubly housed in cages with 24 h access to food and water and were subject to a standard 12 h light/dark cycle. All experiments were performed in accordance with the UK Animals (Scientific Procedures Act) 1986.

### Novel Object Recognition

Experiments took place in an arena (50 × 50 × 50 cm) made of wood with black painted sides. The floor of the arena was painted grey and covered in sawdust. A black curtain surrounded the arena and ensured that the effect of any external stimuli was reduced. The animal was observed using a digital video camera and a monitor. The objects were triplicate copies that varied in size, shape and colour and were made from plastic, glass or metal.

**Fig. 1:**
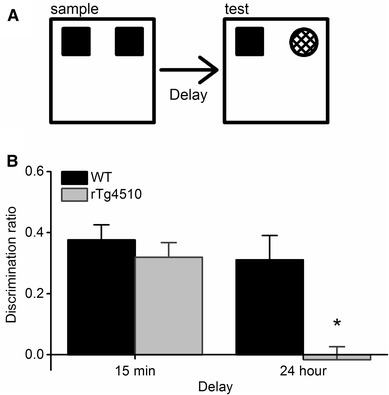
Novel object recognition memory is impaired in rTg4510 mice. **a** Schematic representation of the novel object recognition task. During the sample phase, the mice are placed in a square arena and presented with two identical objects and allowed to freely explore for 10 min. The mice were then removed from the arena and, following a variable delay period of either 15 min or 24 h, were returned to the arena for the test phase, where they were presented with one object identical to the two presented in the sample phase and a novel object. The discrimination ratio was calculated as the time spent exploring the novel object divided by the total exploration time. **b** When a 15 min delay period was imposed, both WT and rTg4510 mice (n = 15 for both groups) were capable of discriminating between the novel and familiar objects, and thus spent significantly more time exploring the novel objects (P < 0.05, one sample t-test). However, when a 24 h delay period was imposed, only the WT mice were able to discriminate between the novel and familiar object (P < 0.05, one sample *t* test), whilst rTg4510 mice could not (P = 0.7, one-sample *t* test). ANOVA revealed a significant main effect of genotype (F_1,28_ = 10.7, P = 0.003) and a significant delay x genotype interaction (F_1,28_ = 6.13, P = 0.02,*)

All experiments took place during the light cycle. Each mouse was habituated to an empty arena for 10 min each day, for 4 days before the experiments began. The discrimination test had a sample/acquisition phase, followed by a delay of either 15 min or 24 h, then a test/choice phase. During the sample phase the animal was placed in the arena and allowed to explore freely two identical copies of an object for 10 min. Exploration of an object was defined as directing the nose of the animal to the object at a distance of < 1 cm. Sitting on an object was not considered exploratory behaviour. The animal was returned to the home cage for the delay period. During the test phase the animals were returned to the arena where there was an identical copy of the object presented in the sample phase and a novel object occupying the same location as the objects in the sample phase. The animal remained in the arena for 5 min while the time spent exploring both objects was recorded (see Fig. 1a for schematic). The objects used in the sample and test phases were counterbalanced. The location of the objects in each phase of the experiment was counterbalanced. Fifteen mice of each genotype were used and each experiment (i.e. delay of 15 min or 24 h) was repeated twice with a separation of 3 days.

Total exploratory times were calculated for the objects used as novel or familiar. The difference in time spent between exploring the novel object versus the familiar object was calculated and expressed as a ratio of total time spent exploring both objects (the discrimination ratio).

### Statistical Analysis

Statistical analyses were performed using SPSS version 19 (IBM). A two-way analysis of variance (ANOVA) was used to assess the effects of genotype and delay time on the discrimination ratio, using a between-subjects design. Post-hoc simple main effects tests were carried out as appropriate. The ability of individual groups to discriminate was determined using a one sample, two tailed *t* test. The alpha level for statistical significance was set at 0.05.

### Slice Electrophysiology

Perirhinal slices were prepared from male rTg4510 and littermate WT mice aged 9–10 months. Mice were euthanized by cervical dislocation and the brain was quickly removed and placed in ice cold (~ 4 °C) sucrose-based cutting solution, containing (in mM): sucrose, 189; d-glucose, 10; NaHCO_3_, 26; KCl, 3; MgCl_2_, 5; CaCl_2_, 0.1; NaH_2_PO_4_, 1.25, continuously bubbled with carbogen (95 O_2_, 5% CO_2_) as described previously [30]. The two hemispheres of the brain were then separated by a midsagittal section and the caudal part of the brain was removed by a single slice at 45° to the dorsoventral axis. Each hemisphere was glued to the vibroslice stage and 400 µm slices were prepared using a vibratome (Leica VT1200). The slices were then transferred to a submerged storage container of artificial cerebrospinal fluid (aCSF) comprised of (in mM): NaCl, 124; KCl, 3; NaHCO_3_, 26; CaCl_2_, 2; NaH_2_PO_4_, 1.25; MgSO_4_, 1; d-glucose, 10, also continuously bubbled with carbogen. The chamber was slowly heated to 32–34 °C for 30 min and then kept at room temperature for a minimum of 1 h. When ready to be used the slices were transferred to the submerged recording chamber that was continuously perfused with aCSF (maintained at ~ 30 °C) at a rate of approx. 2 ml/min.

### Synaptic Plasticity

Field recordings were carried out with a recording electrode placed in cortical layer II/III adjacent to the rhinal sulcus. The recording electrode was a micropipette of borosilicate glass (resistance 3–5 MΩ) filled with aCSF. Field excitatory postsynaptic potentials (fEPSP) were evoked using a concentric bipolar stimulating electrode (inner diameter 12.5 µM; FHC, Bowdoinham, MS, USA) connected to a constant current stimulator box (Digitimer, Welwyn Garden City, UK) placed in layer II, on the temporal side of the rhinal sulcus For each time point the average of four consecutive fEPSP amplitudes was measured and expressed as a percentage of the mean EPSP amplitude during the entire 30 min baseline before the conditioning stimulus. Following a stable baseline the slices were then subject to one of three conditioning stimuli with the intention of evoking LTD: 1 Hz train for 15 min, 5 Hz for 3 min or bath application of 50 µM CCh (Sigma-Aldrich, St Louis, MO) for 20 min. During these conditioning trains, the stimulus strength remained unchanged compared to baseline recordings. The fEPSP amplitude was then recorded for a further 1 h. To determine the cholinergic dependence of the LTD following a 5 Hz stimulation 20 µM Scopolamine hydrobromide (Sigma-Aldrich, St Louis, MO) was bath applied for 20 min prior to and during the conditioning stimulation. To further confirm the cholinergic role in the 5 Hz LTD an acetylcholine esterase inhibitor, Donepezil hydrochloride (Abcam Biochemicals, Cambridge, UK) was bath applied (1 µM) for 20 min prior to and during the conditioning stimulation.

### Synaptic Transmission

Input/output curves were calculated by measuring fEPSP amplitudes following incremental increases in stimulus strength of from 0 to 30 µA. Stimuli were applied every 20 s. A paired pulse profile was calculated by measuring the amplitude ratio in fEPSP pairs evoked at 50% of maximal stimulation strength. The inter-stimulus intervals examined were 10, 18, 32, 56, 100, 180, 320, 560 ms and 1 s.

### Data Acquisition and Statistical Analysis

Data were recorded using an Axopatch 200B amplifier (Molecular Devices, Sunnyvale, CA) in series with a secondary instrumentation amplifier (NPI electronics GmbH, Tamm, Germany). Recordings were lowpass filtered (10 kHz), highpass filtered (1 Hz) and digitized (50 Hz) using a 1322A Digidata (Molecular Devices, Sunnyvale, CA) and pClamp 10 acquisition software (Molecular Devices, Sunnyvale, CA, USA). Data were reanalyzed offline using Clampfit 10.3 (Molecular Devices, Sunnyvale, CA) and custom written scripts in Matlab (Mathworks, Natwick, MA). Data pooled across slices are expressed as the mean ± SEM, effects of the conditioning stimulation were measured at 50–60 min after the induction. Statistical significance was determined using an appropriate parametric test with an alpha value of 0.05.

## Results

Visual recognition memory is disrupted in patients suffering from dementias [2] and has been reported to be disrupted in a different transgenic mouse model of tauopathy and when mutant tau is delivered virally to PRh [28]. To explore whether this form of memory is similarly disturbed in rTg4510 mice, we examined novel object discrimination in 8–9 month old subjects alongside aged-matched, wildtype littermate controls (WT). Following the shorter retention delay of 15 min both WT and rTg4510 mice successfully discriminated between the novel and familiar objects. Thus, both the WT (n = 15) and rTg4510 (n = 15) groups had mean discrimination ratios significantly greater than zero (0.38 ± 0.05, t_14_ = 7.6, P < 0.01 and 0.32 ± 0.05, t_14_ = 6.7, P < 0.01, respectively; one sample t-test for both groups; Fig. 1b). In contrast, when mice were subjected to a longer delay (24 h) between sample and test phase, the WT mice were still able to discriminate between the novel and familiar objects (and thus had a mean discrimination ratio significantly greater than zero: 0.31 ± 0.08; t_14_ = 3.9, P < 0.01 one-sample *t* test; Fig. 1b), however the rTg4510 mice were unable to discriminate between novel and familiar objects (mean discrimination ratio: − 0.02 ± 0.04; t_14_ = − 0.4 P = 0.7, one-sample *t* test; Fig. 1b). Using a, between-subjects ANOVA we found that there was a significant main effect of genotype (F_1,56_ = 11.5, P = 0.001) and a significant main effect of delay (F_1,56_ = 12.5, P = 0.001). Furthermore, there was also a significant delay time x genotype interaction (F_1,56_ = 5.7, P = 0.02). A post-hoc analysis revealed that there was a significant difference between discrimination ratios of the WT and rTg4510 groups when a 24 h delay (P < 0.01) was used, but not when a 15 min delay was used (P = 0.4). There was also a significant difference between the 15 min and 24 h delay discrimination ratios in the rTg4510 group (P < 0.01), but not the WT group (P = 0.5; Fig. 1b). Importantly, there was no significant main effect of genotype (F_1,56_ = 0.08, P = 0.8) or delay (F_1,56_ = 0.06, P = 0.8), nor was there an genotype x delay interaction (F_1,56_ = 0.08, P = 0.8) on total object exploration time during the sample phase (Table 1). Likewise, during the test phase, there was no significant main effect of genotype (F_1,59_ = 3.5, P = 0.1) or delay (F_1,59_ = 0.3, P = 0.6), nor was there an genotype x delay interaction (F_1,59_ = 0.7, P = 0.4) on total object exploration time (Table 1). Taken together these data suggest that rTg4510 mice were able to support short-term visual recognition memory (i.e. with a 15 min delay between sample and test phase), however, when the delay period was extended to 24 h rTg4510 mice were unable to distinguish previously experienced objects.

**Table 1:** Mean exploration times (± S.E.M.) in the sample and test phases of the novel object recognition task

Genotype	Delay	Exploration in sample phase (s)	Exploration in test phase (s)
WT	15 min	86.2 ± 6.3	34.7 ± 2.9
	24 h	82.2 ± 9.0	27.5 ± 3.9
rTg4510	15 min	81.6 ± 15.8	40.4 ± 8.4
	24 h	82.0 ± 13.8	42.2 ± 5.0

Synaptic plasticity, and in particular LTD, in the PRh has been identified as a cellular mechanism underlying visual recognition memory [17]. By altering the LTD-induction protocol, different forms of LTD, dependent on different neurotransmitter receptor systems, can be evoked in PRh. For example, 1 Hz stimulation for 15 min results in an NMDA-receptor dependent form of LTD in slices prepared from young rats [16]. Interestingly, this form of LTD cannot be elicited in slices prepared from adult (8–10 week old) wildtype mice but can be evoked in transgenic mutant APP-overexpressing CRND8 counterparts [31]. To determine whether a similar LTD phenotype can be observed in our model of tauopathy, we made extracellular field potential recordings from layer II/III of PRh slices prepared from 8 to 9 month old rTg4510 and WT littermate controls.

**Fig. 2:**
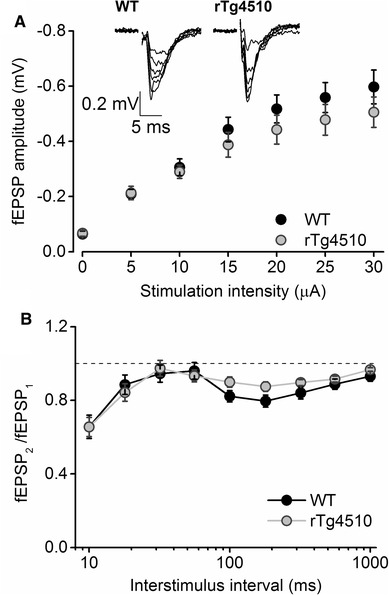
Basal excitatory synaptic transmission and short term plasticity were unaffected by tau_P301L_ overexpression. **a** Pooled input–output relationships showing the mean (± S.E.M.) amplitude of fEPSPs recorded from layer II/III of the PRh in response to increasing stimulus intensities. No significant difference in input–output relationships were observed between WT (n = 13) and rTg4510 slices (n = 9; F_1,20_ = 0.8, P = 0.4). Inset traces are example fEPSPs evoked by a range of stimulus strengths recorded from a WT and a rTg4510 slice. In these, and all subsequent electrophysiological traces, the stimulus artefact has been removed for clarity. **b** Graph depicts the paired pulse ratio, calculated as the amplitude of 2nd fEPSP divided by the amplitude of the 1st fEPSP (fEPSP_2_/fEPSP_1_), at a range of inter-stimulus intervals (10–1000 ms). No significant difference in the paired pulse profile between WT (n = 17) and rTg4510 (n = 16) synaptic responses could be detected (F_1,31_ = 0.39, P = 0.5)

Synaptic transmission is known to be disturbed in the hippocampus of many APP- [32–35] and tau-based [23] mouse models of dementia. To explore whether a similar disruption occurs in PRh synaptic microcircuits of rTg4510 mice, we constructed input–output curves for PRh synaptic responses, by varying the current passed through the stimulating electrode. Increasing the current amplitudes resulted in larger amplitude fEPSPs in both genotypes. However there was no significant difference between the input–output relationships recorded from WT or rTg4510 slices (Fig. 2a). Thus, the mean amplitude of responses evoked from WT slices (with a stimulus intensity of 30 µA) was 0.60 ± 0.06 mV (n = 13 slices from 7 animals) whilst in rTg4510 slices it was 0.51 ± 0.06 mV (n = 9 slice from 5 animals). A repeated measures, between-subjects ANOVA revealed no significant main effect of genotype (F_1,20_ = 0.8, P = 0.4), suggesting that basal synaptic transmission in the PRh is relatively unaffected by transgenic overexpression of mutant tau protein. This contrasts with electrophysiological observations in the P301S mouse line [28] in which anatomically more widespread mutant tau expression is present.

We next sought to establish whether short-term synaptic plasticity was altered in the PRh of rTg4510 mice. Paired stimuli delivered at a range of intervals (between 10 and 1000 ms) resulted in a bi-phasic paired pulse profile in WT slices (Fig. 2b). At short intervals (10 ms) the fEPSPs exhibited substantial (~ 30–40%) paired-pulse depression, a phenomenon that was largely absent at intervals between 30 and 60 ms and then reappeared at intervals between 100 and 600 ms. These data suggest the existence of at least two mechanisms underlying short-term plasticity of fEPSPs in PRh. Nevertheless, no significant differences in paired-pulse profiles were observed between WT (n = 17 slices from 9 animals) and rTg4510 (n = 16 slices from 7 animals) PRh slices (main effect of genotype: F_1,31_ = 0.39, P = 0.5; Fig. 2b).

**Fig. 3:**
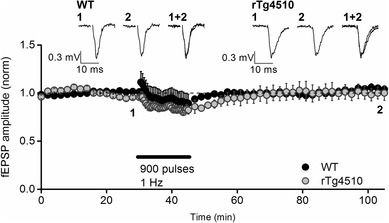
LTD could not be induced using a 1 Hz stimulation paradigm in either WT or rTg4510 PRh slices. The graph depicts the pooled data from 5 WT and 5 rTg4510 slices showing the response to 900 stimuli delivered at 1 Hz (represented by the black bar). No significant LTD (1 h post stimulation) was induced in either genotype (P > 0.05, paired 2-sample *t* tests in both groups). Furthermore, there was no significant difference in the post-stimulation baseline normalised fEPSP amplitudes between WT and rTg4510 mice (P = 0.5, unpaired 2 sample *t* test). Inset traces are averages of 4 consecutive synaptic responses from an example experiment in both groups, taken from the time points indicated on the graph

To study LTD in the PRh, we first employed a stimulation paradigm that, in juvenile rats, has been shown to elicit an NMDA receptor-dependent form of LTD [16]. Synaptic responses were first evoked at low frequency (0.033 Hz) for 30 min to ensure stability and were subsequently stimulated at 1 Hz for 15 min. Neither WT nor rTg4510 slices exhibited significant long term synaptic plasticity in response to this stimulation paradigm (Fig. 3). Thus, fEPSP amplitudes were 105.3 ± 2.7% of baseline (n = 5 slice from 5 animals, t_4_ = 1.8, P = 0.1, paired 2-sample *t* test) in WT slices and 101.1 ± 5.4% of baseline in rTg4510 slices (n = 5 slices from 5 animals, t_4_ = 0.2, P = 0.8, paired 2-sample t-test) 1 h after the 1 Hz stimulation protocol. Furthermore, there was no significant difference between the levels of post-stimulation plasticity in WT and rTg4510 mice (t_8_ = 0.7, P = 0.5, unpaired 2 sample *t* test; Fig. 3).

**Fig. 4:**
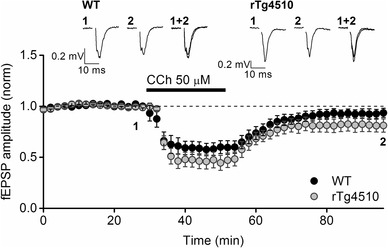
LTD induced by bath application of carbachol (CCh) is unaffected by overexpression of tau_P301L_. The graph depicts the pooled data from 8 WT and 8 rTg4510 slices showing the response to a 20 min bath application of a high concentration (50 µM) of the mAChR agonist CCh (represented by the black bar). Both WT and rTg4510 slices exhibited significant levels of LTD 40 min after wash-out of the agonist (P < 0.05, paired t-tests, both groups). However, there was no significant difference in the magnitude of the LTD between the two groups (P = 0.4, unpaired t-test). Inset traces are averages of 4 consecutive synaptic responses from an example experiment in both groups, taken from the time points indicated on the graph

Activation of muscarinic acetylcholine receptors (mAChRs) is known to induce a form of chemical LTD in the perirhinal cortex [36, 37] which is thought to activate cellular mechanisms similar to those activated during recognition memory [38]. Application of the muscarinic agonist carbachol (CCh; 50 µM) for 20 min, transiently suppressed the synaptic responses in both WT and rTg4510 PRh slices by 40–50% (Fig. 4). After the agonist was washed out, the fEPSPs amplitudes increased in amplitude but stabilised at a level significantly lower than the basal fEPSPs, in both genotypes. Thus, 40 min after the washout of CCh, fEPSPs in WT slices were 89.9 ± 0.4% of baseline amplitudes (n = 8 slices from 8 animals, t_7_ = − 2.4, P = 0.04, paired t-test) whilst in rTg4510 slices, fEPSP amplitudes were 85.3 ± 0.04% of baseline (n = 8 slices from 5 animals, t_7_ = − 3.8, P = 0.007, paired *t* test). A direct comparison of the levels of CCh-induced LTD revealed that there was no significant difference in between WT and rTg4510 slices (t_14_ = 0.8, P = 0.4, unpaired *t* test).

**Fig. 5:**
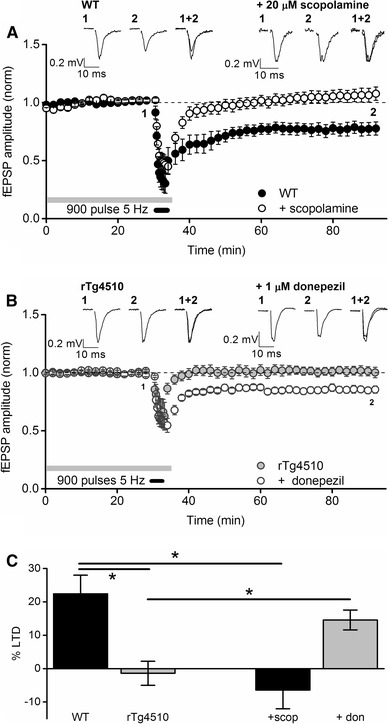
Deficits in mAChR-dependent synaptic LTD in rTg4510 PRh slices. **a** The graph shows pooled data from WT slices under control conditions (n = 7) or pretreated with 20 µM scopolamine (n = 7; grey bar). In control slices, 900 stimuli delivered at 5 Hz (represented by the black bar) resulted in a robust and significant LTD of synaptic transmission in the PRh (P = 0.008, paired *t* test) which was absent when the stimuli were delivered in the presence of scopolamine (P = 0.6, paired *t* test). **b** The graph shows pooled data from rTg4510 slices under control conditions (n = 9) or pretreated with 1 µM donepezil (n = 7; grey bar). In control rTg4510 slices the same 5 Hz stimulation paradigm as delivered in A could not induce significant levels of LTD of synaptic transmission in the PRh (P = 0.67, paired *t* test). However, in the presence of the acetylcholinesterase inhibitor donepezil, significant levels of LTD were observed (P = 0.002, paired *t* test). The inset traces in A and B are averages of 4 consecutive synaptic responses from example experiments in each group, taken from the time points indicated on the graphs. **c** Summary bar chart depicting the significant deficit in 5 Hz-induced PRh LTD in rTg4510 slices when compared with WT littermates (left hand bars; *P < 0.05, unpaired 2-sample *t* tests). On the right hand side of this graph, for comparison, are the levels of LTD in WT slices in the presence of scopolamine and rTg4510 slices in the presence of donepezil

Chemical LTD protocols, such as that described above, can be useful models to explore cellular mechanisms, however, since they rely on bath application of high concentrations of synthetic agonists, they do not readily replicate the physiological stimulation paradigms which induce LTD in vivo. In this regard, an electrical stimulation protocol, consisting of 900 stimuli delivered at 5 Hz for 3 min, can elicit a form of LTD dependent on activation of mAChRs [15]. Therefore, we examined the properties of this form of mAChR-dependent LTD in rTg4510 PRh slices. Stimulation of synaptic inputs into the PRh at 5 Hz for 3 min resulted in a robust and significant LTD in WT slices which was still apparent 60 min post stimulation (fEPSP amplitude 77.6 ± 5.6% baseline, n = 7 slice from 7 animals, t_6_ = − 4.0, P = 0.008, paired *t* test; Fig. 5a, c). Preincubation of WT slices in the mAChR antagonist scopolamine (20 µM) inhibited the induction of LTD using this stimulation pattern, suggesting a crucial role for mAChR activation in the induction of this form of LTD. Thus, 1 h after 5 Hz stimulation in the presence of scopolamine, the mean fEPSP amplitude was 106.4 ± 5.6% of baseline (n = 7 slice from 7 animals, t_6_ = 1.3, P = 0.3, paired *t* test), significantly different from levels of LTD in untreated slice (t_12_ = − 3.5, P = 0.005, unpaired 2 sample *t* test; Fig. 5a, c). Interestingly, rTg4510 slices could not express this form of mAChR-dependent LTD. Thus, 1 h after the 5 Hz stimulation paradigm, the mean fEPSP amplitude was 101.2 ± 4.1% of baseline, not significantly different from baseline levels (n = 8 slice from 7 animals, t_7_ = 0.6, P = 0.6, paired *t* test) and significantly different from WT levels of plasticity (t_13_ = − 3.4, P = 0.005, unpaired 2-sample *t* test; Fig. 5b, c).

Given the role for mAChRs in the induction of this form of LTD, we reasoned that we may be able to rescue the deficit in perirhinal LTD by boosting the cholinergic systems. To test this hypothesis, we bath applied 1 µM of the cholinesterase inhibitor donepezil to rTg4510 perirhinal slices at least 20 min prior to delivery of the 5 Hz stimulation train. Interestingly, under these conditions we found that 5 Hz stimulation was sufficient to induce LTD in the rTg4510 slices, such that 1 h after stimulation the fEPSP was significantly smaller than baseline levels (84.9 ± 3% of baseline, n = 7 slices from 7 animals, t_6_ = − 4.2 P = 0.006, paired *t* test). Furthermore, the level of LTD was significantly greater than that induced in untreated rTg4510 slices (t_13_ = − 3.1, P = 0.008, unpaired t-test; Fig. 5b, c).

## Discussion

The present study investigated PRh-dependent visual recognition memory and synaptic plasticity in a mouse model of tauopathy. Our data suggest that forebrain overexpression of mutant tau protein interferes with long term consolidation of visual recognition memory, such that 24 h after being exposed to an object, rTg4510 mice could not discriminate between this familiar object and a novel object. This disruption to long term visual recognition memory was correlated with a profound deficit in mAChR-dependent LTD in the PRh, which was ameliorated by preapplication of donepezil, an acetylcholinesterase inhibitor.

Short-term visual recognition memory (i.e. when mice were subjected to a 15 min delay between sample and test phase) was not significantly affected by tau_P301L_ overexpression. This is important since it suggests that rTg4510 mice were still capable of visually discriminating between the objects and retaining that information over short time periods. Therefore, the deficit observed when a longer (24 h) delay was imposed, was not due to deterioration of the visual system, either at the central or peripheral level. Rather, the deficit observed with a 24 h delay likely resulted from a failure of long term memory systems, probably associated with the PRh [5]. These findings are similar to those reported by other groups. A study in rTg4510 mice found that at short- (5 min), but not longer- (15 min), intervals between the sample and test phases, the tauopathy animals were able to discriminate between familiar objects in novel locations [39]. This task employed by Crimins et al. [39] was cognitively more demanding than the task employed here (i.e. multiple objects were used in multiple, shorter sample phases) and is thought to involve more complex network interactions between multiple associational brain areas [9]. This difference in the paradigms used may explain why, in our study, rTg4510 mice were able to perform as well as WT mice when a 15 min delay between sample and test phase was employed. Object recognition studies performed within a Y shaped arena using both homozygous and heterozygous P301S mice (both of which develop a more widespread tau pathology than rTg4510 mice) have also identified deficits at long inter-trial intervals (3 and 24 h) but not at 1 min. These deficits were also shown to be absent in younger pre-symptomatic P301S animals—although the age lacking an effect in the heterozygotes varied between the two studies [28, 29]. This group have also produced deficits in their version of task using viral expression of P301S tau locally in the perirhinal cortex and can afford a degree of phenotypic rescue by manipulating the perineuronal extracellular matrix [28, 29].

Long term depression of synaptic transmission in the PRh is thought to underlie visual recognition memory [17, 18]. There are multiple forms of LTD in the PRh generated by activating different neurotransmitter receptor systems. For example, pharmacological blockade of mAChRs by scopolamine has been shown to inhibit LTD induced by 5 Hz stimulation trains in PRh as well as interfering with object recognition memory. Alternatively, stimulation at lower frequencies (1 Hz) has been reported to induce a form of LTD which is sensitive to NMDA receptor blockade [16]. Our data suggest that PRh slices prepared from 8 to 9 month old WT mice are capable of supporting mAChR-mediated, but not NMDA receptor-mediated, LTD. The latter finding agrees with a recently published study which showed that PRh slices from adult WT mice did not express LTD in response to a 1 Hz stimulation paradigm [31]. In contrast, in this same study, Romberg et al. showed that slices prepared from CRND8 mice, which overexpress a mutant form of APP and thus accumulate an Aβ peptide pathology, exhibited an NMDA receptor-mediated aberrant form LTD, which the authors attribute to a form of ‘false recognition memory’ [31]. We also found that PRh slices prepared from mature adult WT mice did not express LTD in response to a 1 Hz stimulation paradigm, however, neither did the rTg4510 slices. It is likely therefore, that the deficits in visual recognition memory observed in response to mutant tau overexpression differ mechanistically from the deficits seen when mutant APP is overexpressed. This perhaps highlights a wider dichotomy between these two types of models of neurodegenerative pathologies which should be borne in mind when trying to draw biological inference from transgenic mouse models of disease.

It should be noted that an LTD-like plasticity response has been observed in a neurophysiological study of a mouse model of tauopathy [28]. In this work there was no difference in perirhinal LTD when WT and P301S animals were compared. The reasons why LTD was seen in this study of P301S mice but not in ours may reflect methodological differences; these include the use of much younger mice (3 Mo), a bath solution with higher K^+^ concentration (which would cause depolarization) and much lower bicarbonate. Importantly, the LTD induction protocol employed was different as it involved 900 paired stimulations (20 ms interstimulus interval), so 1800 synaptic activations in total. It is possible the nature of the conditioning stimulus may recruit sufficient extra cholinergic drive to generate a form of plasticity; in the hippocampus, for example, it is reported that low frequency trains of paired pulses are required to induce LTD [40].

The deficit in mAChR-dependent LTD was at least partially reversed by bath application of the acetylcholinesterase donepezil (marketed under the trade name Aricept and licensed for the treatment of mild-to-moderate Alzheimer’s disease), suggesting that the LTD deficit was related to dysfunction in the cholinergic system. Furthermore, bath application of the muscarinic agonist CCh was sufficient to induce mAChR-dependent LTD in rTg4510 PRh slices. Taken together these data suggest that the molecular mechanisms underlying LTD (thought to be mainly postsynaptic) [17] remain largely intact. Nevertheless, it is likely that there is a loss or reduction of cholinergic innervation of the PRh in rTg4510 mice, such that when the 5 Hz stimulation protocol is delivered, insufficient levels of acetylcholine are released to induce LTD. A similar deficit in LTD induced by 5 Hz stimulation of PRh slices prepared from Tg2576 (overexpressing APP with the Swedish mutation) has been reported previously [41], raising the possibility that this model also has cholingeric deficits which contribute to novel object recognition impairments.

The cholinergic system has, for many years, been known to degenerate in dementias such as AD and FTD [42, 43] and as a result most of the currently available treatments for dementia are acetylcholinesterase inhibitors. Indeed, modulation of the cholinergic receptor system is still an area of intense drug development activity [44]. Acetylcholinesterases are capable of enhancing deficient recognition memory in cognitively impaired individuals [45], suggesting that cholinergic dysfunction may contribute to these types of functional deficits. Potentially, therefore, impairments to PRh synaptic LTD may play a critical role in diseases affecting cognition such as AD and FTD. Therefore, novel therapeutic agents which positively modulate PRh LTD may be useful in the treatment of dementia.
